# Systemic hypothermia increases PAI-1 expression and accelerates microvascular thrombus formation in endotoxemic mice

**DOI:** 10.1186/cc5074

**Published:** 2006-10-24

**Authors:** Nicole Lindenblatt, Michael D Menger, Ernst Klar, Brigitte Vollmar

**Affiliations:** 1 Department of Experimental Surgery, University of Rostock, Schillingallee, Rostock 18055, Germany; 2 Department of General Surgery, University of Rostock, Schillingallee, Rostock, 18055, Germany; 3 Institute for Clinical and Experimental Surgery, University of Saarland, Kirrberger Straße, Homburg-Saar, 66424, Germany

## Abstract

**Introduction:**

Hypothermia during sepsis significantly impairs patient outcome in clinical practice. Severe sepsis is closely linked to activation of the coagulation system, resulting in microthrombosis and subsequent organ failure. Herein, we studied whether systemic hypothermia accelerates microvascular thrombus formation during lipopolysacharide (LPS)-induced endotoxemia *in vivo*, and characterized the low temperature-induced endothelial and platelet dysfunctions.

**Methods:**

Ferric-chloride induced microvascular thrombus formation was analyzed in cremaster muscles of hypothermic endotoxemic mice. Flow cytometry, ELISA and immunohistochemistry were used to evaluate the effect of hypothermia on endothelial and platelet function.

**Results:**

Control animals at 37°C revealed complete occlusion of arterioles and venules after 759 ± 115 s and 744 ± 112 s, respectively. Endotoxemia significantly (*p *< 0.05) accelerated arteriolar and venular occlusion in 37°C animals (255 ± 35 s and 238 ± 58 s, respectively). This was associated with an increase of circulating endothelial activation markers, agonist-induced platelet reactivity, and endothelial P-selectin and plasminogen activator inhibitor (PAI)-1 expression. Systemic hypothermia of 34°C revealed a slight but not significant reduction of arteriolar (224 ± 35 s) and venular (183 ± 35 s) occlusion times. Cooling of the endotoxemic animals to 31°C core body temperature, however, resulted in a further acceleration of microvascular thrombus formation, in particular in arterioles (127 ± 29 s, *p *< 0.05 versus 37°C endotoxemic animals). Of interest, hypothermia did not affect endothelial receptor expression and platelet reactivity, but increased endothelial PAI-1 expression and, in particular, soluble PAI-1 antigen (sPAI-Ag) plasma levels.

**Conclusion:**

LPS-induced endotoxemia accelerates microvascular thrombus formation *in vivo*, most probably by generalized endothelial activation and increased platelet reactivity. Systemic hypothermia further enhances microthrombosis in endotoxemia. This effect is associated with increased endothelial PAI-1 expression and sPAI-Ag in the systemic circulation rather than further endothelial activation or modulation of platelet reactivity.

## Introduction

Microvascular thrombus formation with subsequent microvessel occlusion and hypoperfusion is a major contributor to organ dysfunction during sepsis [1]. It is well recognized that sepsis involves a complex interaction between the inflammatory and the coagulation system [2]. Bacterial endotoxin (lipopolysacharide (LPS)) induces a variety of metabolic, cellular and regulatory effects that are accompanied by fever in mammals [3]. The pyrogenic effects are exerted by increasing the production of endogenous cytokines such as IL-1, IL-6 and tumor necrosis factor (TNF)-alpha. Severe sepsis is almost invariably associated with activation of the coagulation system, potentially resulting in disseminated intravascular coagulation. Together with other components, the tissue factor-driven generation of thrombin with fibrin accumulation and platelet activation play a pivotal role in this setting [4]. In sepsis, both the coagulation and the fibrinolytic system may be affected, as indicated by decreased activation of thrombomodulin and protein C as well as reduction of anti-fibrinolysis and enhancement of plasminogen activator inhibitor (PAI)-1 expression [2]. The production of procoagulant factors, as well as their interaction with platelets and leukocytes in the microvasculature, may lead to intravascular fibrin formation [5].

Septic patients, who develop hypothermia during the course of the illness, have a significantly worse prognosis compared to those who develop fever or maintain body temperature. In addition, in animal models of sepsis it has been observed that hypothermia is associated with immune dysfunction and an unfavorable outcome [6,7]. Presently, it is not clear whether hypothermia during severe sepsis merely serves as a surrogate marker for progression of the disease, representing a general failure of regulatory functions, or whether hypothermia itself negatively influences the course of the disease. Additionally, the reasons for the worse prognosis during sepsis with hypothermia have not been clearly identified.

In previous experiments we were able to show that hypothermia accelerates microvascular thrombus formation and increases platelet reactivity [8]. Based on these studies we hypothesize that hypothermia during severe sepsis aggravates the already existing procoagulant state. This may lead to a further aggravation of microvascular thrombus formation, possibly representing a cause of the worse outcome in septic patients with hypothermia in clinical practice. To address this issue, we analyzed the kinetics of microvascular thrombus formation in a murine *in vivo *LPS model of systemic hypothermia at 34°C and 31°C. The effects of endotoxemia and hypothermia on endothelial function were further determined by assessing plasma levels and tissue expression of endothelial activation markers. We additionally evaluated hypothermia-induced platelet response *in vitro *using temperatures of 34°C and 31°C, which are likely to be encountered during severe hypothermia in the setting of prolonged sepsis.

## Materials and methods

### Mouse cremaster muscle preparation

Upon approval by the local government, all experiments were carried out in accordance with the German legislation on protection of animals and the National Institutes of Health 'Guide for the Care and Use of Laboratory Animals' (Institute of Laboratory Animal Resources, National Research Council). Male C57BL/6J mice with a body weight (bw) of 20 to 25 g were anesthetized by an intraperitoneal injection of ketamine (90 mg/kg bw) and xylazine (25 mg/kg bw) and a polyethylene catheter was placed into the right jugular vein, serving for application of fluorescent dyes.

For the study of microvascular thrombus formation, we used the cremaster muscle preparation as originally described by Baez in rats [9] and applied by our group in mice [8,10]. Before preparation of the cremaster muscle, animals were placed on a heating pad coupled to a rectal probe. A midline incision of the skin and fascia was made over the ventral aspect of the scrotum and extended up to the inguinal fold and to the distal end of the scrotum. The incised tissues were retracted to expose the cremaster muscle sac, which was maintained under gentle traction to carefully separate the remaining connective tissue by blunt dissection from around the cremaster sac. The cremaster muscle was then incised without damaging larger anastomosing vessels. Hemostasis was achieved with 5–0 threads serving also to spread the tissue. After dissection of the vessel connecting the cremaster and the testis, the epididymus and testis were put to the side of the preparation. The preparation was performed on a transparent pedestal to allow microscopic observation of the cremaster muscle microcirculation by both transillumination and epi-illumination techniques.

After surgical preparation, the animals were allowed to recover for 15 minutes. Thrombus formation was then induced in randomly chosen venules (*n *= 1 to 2 per preparation) and arterioles (*n *= 1 to 2 per preparation).

### Experimental design

Mice were pretreated with LPS (*Escherichia coli*, serotype 0128:B12; LOT# 069H4097, Sigma-Aldrich, Munich, Germany) at a dose of 10 mg/kg intraperitoneally (ip) 24 hours before the beginning of the experiments. Following induction of anesthesia, animals were placed on a customized platform with an incorporated heating pad to facilitate microscopy of the cremaster muscle. Temperature was controlled by a rectal probe and maintained at 37°C, 34°C or 31°C. Animals pretreated with physiological saline (10 ml/kg bw, -24 h ip) with a body core temperature of 37°C served as controls. Overall, 10 saline/37°C control cremaster muscles (*n *= 5 animals) and eight cremaster muscles of each of the LPS/37°C, LPS/34°C and LPS/31°C groups (*n *= 4 animals for each group) were studied. Different animals were committed to the analyses at the three different temperatures.

The assumption that the rectal temperature equaled the core body temperature was confirmed by additional experiments using a LICOX probe (LICOX 1, GMS, Kiel-Mielkendorf, Germany) as described before [8]. Depending on the rectal temperature at the beginning of the experiment and the desired final temperature, heating was started immediately or after the animal cooled down to the required temperature. Artificial cooling was not necessary, because most of the animals displayed a considerable drop in body temperature after induction of anesthesia. After the appropriate temperature, according to randomization of animals, was reached and remained stable for at least 30 minutes, the preparation was started and animals were allowed to recover from the surgical trauma for 15 minutes. Thrombus formation was then induced in randomly chosen venules (*n *= 1 to 2 per preparation) and arterioles (*n *= 1 to 2 per preparation), as described in the next section. Animals were kept under the respective temperature conditions during the whole course of the experiment, including intravital microscopy and microvascular thrombus induction.

### *In vivo *thrombosis model

After intravenous injection of 0.1 ml 5% fluorescein isothiocyanate-labeled dextran (MW 150000, Sigma-Aldrich, Munich, Germany) and subsequent circulation for 30 s, the cremaster muscle microcirculation was visualized by intravital fluorescence microscopy using a Zeiss microscope (Axiotech vario, Zeiss, Jena, Germany). The microscopic procedure was performed at a constant room temperature of 21 to 23°C. The epi-illumination setup included a 100 W HBO mercury lamp and a blue filter (450 to 490 nm/>520 nm excitation/emission wavelength). Microscopic images were recorded by a charge-coupled device video camera (FK 6990A-IQ, Pieper, Schwerte, Germany) and stored on videotapes for off-line evaluation (S-VHS Panasonic AG 7350-E, Matsushita, Tokyo, Japan). Using a ×20 water immersion objective (Achroplan x20/0.50W, Zeiss) baseline blood flow was monitored in individual arterioles (diameter range 30 to 50 μm) and venules (diameter range 60 to 80 μm). Thereafter, microvascular thrombosis was induced by spreading of 25 μl ferric chloride solution (12.5 mmol/l; Sigma) over the cremaster muscle every minute, resulting in a continuous superfusion of the tissue [11,12]. Complete vessel occlusion was assumed to have occurred when blood flow ceased for more than 60 s due to thrombotic occlusion. As rapid spreading of ferric chloride solution allowed the study of only one or two arterioles and venules within each preparation, both left and right cremaster muscles of each animal were prepared for analysis of thrombotic vessel occlusion.

Analysis included the time period until sustained cessation of blood flow due to complete vessel occlusion as well as the determination of vessel diameter and blood cell velocity prior to thrombus induction. Vascular wall shear rates were calculated based on the Newtonian definition γ = 8 × V/D, with V representing the red blood cell centerline velocity divided by 1.6 according to the Baker-Wayland factor [13] and D representing the individual inner vessel diameter.

### ELISA of circulating endothelial markers

At the end of each experiment, blood was withdrawn from the inferior vena cava by direct puncture into EDTA syringes, followed by centrifugation (GS-6R Centrifuge, Beckman Coulter, Fullerton, CA, USA) at 200 × g and room temperature for 10 minutes with subsequent storage of plasma at -20°C. Plasma concentrations of circulating, that is, soluble (s)P-selectin, sE-selectin, intercellular adhesion molecule (sICAM)-1, vascular cell adhesion molecule (sVCAM)-1 and plasminogen activator inhibitor-1 antigen (sPAI-Ag) were determined using the respective enzyme immunoassay kits (R&D Systems, Minneapolis, MN, USA, and Molecular Innovations Inc., Southfield, MI, USA).

### Histology and immunhistochemistry

At the end of each experiment, the cremaster muscle was fixed in 4% phosphate buffered formalin for two to three days and embedded in paraffin. From the paraffin-embedded tissue blocks, 4 μm-sections were cut and stained with hematoxylin and eosin for histological analysis. For immunohistochemical demonstration of P-selectin and PAI-1 expression, sections collected on poly-L-lysine-coated glass slides were treated by microwave for antigen unmasking. Goat anti-human P-selectin and goat anti-human PAI-1 (each 1:100; Santa Cruz Biotechnology, Heidelberg, Germany) were used as primary antibodies and incubated for 90 to 120 minutes at room temperature. This was followed by a horseradish peroxidase-conjugated donkey anti-goat antibody (1:25; Santa Cruz Biotechnology) and development using DAB substrate as chromogen. The sections were counterstained with hematoxylin and examined by light microscopy (Zeiss Axioscop 40, Zeiss).

### Preparation of murine platelet rich plasma

For *in vitro *testing of platelet function additional animals were exposed to LPS according to the experimental protocol (10 mg/kg ip; -24 h). Controls received physiological saline (10 ml/kg ip; -24 h). Then 0.5 to 1 ml blood was drawn from the retro-orbital venous plexus with 1.5 cm glass capillaries and collected into a tube containing TRIS buffered saline/heparin (20 U/ml). The sample was centrifuged for five minutes at 500 × g yielding platelet rich plasma that was centrifuged again for eight minutes at 300 × g and 0.5 μM prostacyclin (PGI_2_) was added. The platelet pellet was resuspended and apyrase and Tyrode's buffer were added and centrifugation steps were continued as described elsewhere [14]. Aliquots of platelet suspensions were transferred into a 37°C water bath for 30 minutes of resting to eliminate isolation-induced platelet activation.

Platelet suspensions from LPS-treated animals were incubated for 30 minutes in water baths maintaining temperatures at either 37°C, 34°C or 31°C followed by exposure to thrombin (20 U/ml) and incubation with saturating amounts of the appropriate antibody. Platelets from control animals were kept at 37°C continuously. Platelet suspensions were kept for an additional 30 minutes in the respective covered water baths.

### Flow cytometric analysis of P-selectin, glycoprotein IIb-IIIa and CD107a expression

For evaluation of receptor expression under resting conditions, 5 μl of specific rat anti-mouse P-selectin, glycoprotein (GP)IIb-IIIa (Emfret Analytics, Eibelstadt, Germany), CD107a (BD Biosciences, Heidelberg, Germany) or negative control antibodies and 25 μl platelet suspension were combined and incubated for 15 minutes at room temperature. The reaction was stopped by addition of 400 μl phosphate buffered saline. Analysis was performed within the subsequent 30 minutes. In addition, the same set of experiments was carried out following exposure to thrombin for maximal platelet activation (20 U/ml).

A FACScan flowcytometer (Becton Dickinson, Heidelberg, Germany) was calibrated with fluorescent standard microbeads (CaliBRITE Beads, Becton Dickinson) for accurate instrument setting. Platelets were identified by their characteristic forward and sideward light scatter and selectively analyzed for their fluorescence properties using the CellQuest program (Becton Dickinson) with assessment of 20,000 events per sample. The relative fluorescence intensity of a given sample was calculated by subtracting the signal obtained when cells were incubated with the isotype specific control antibody from the signal generated by cells incubated with the test antibody.

### Statistical analysis

After proving the assumption of normality and equal variance across groups, differences between groups were assessed using one-way analysis of variance (ANOVA) followed by the appropriate *post hoc *comparison test. All data were expressed as means ± standard error of the mean and overall statistical significance was set at *p *< 0.05. Pearson product moment correlation was performed to evaluate significant correlations between parameters of platelet activation and temperature. Statistics and graphics were performed using the software packages SigmaStat and SigmaPlot (Jandel Corporation, San Rafael, CA, USA).

## Results

### Intravital microscopic analysis of microvascular thrombosis

In endotoxemic animals, red blood cell velocities were significantly lower when compared with those of the control group at 37°C (Table 1), indicating compromise of microvascular flow conditions at the beginning of the experiments owing to the endotoxemic state. However, wall shear rates did not differ significantly between the experimental groups. After induction of anesthesia the average core temperature for all animals was 36.7 ± 0.5°C. Body temperature decreased within two to five minutes in the anesthetized animals and reached the desired temperatures of 34°C and 31°C without artificial cooling. In control animals this effect was prevented by warming on a heating plate.

**Figure 1:**
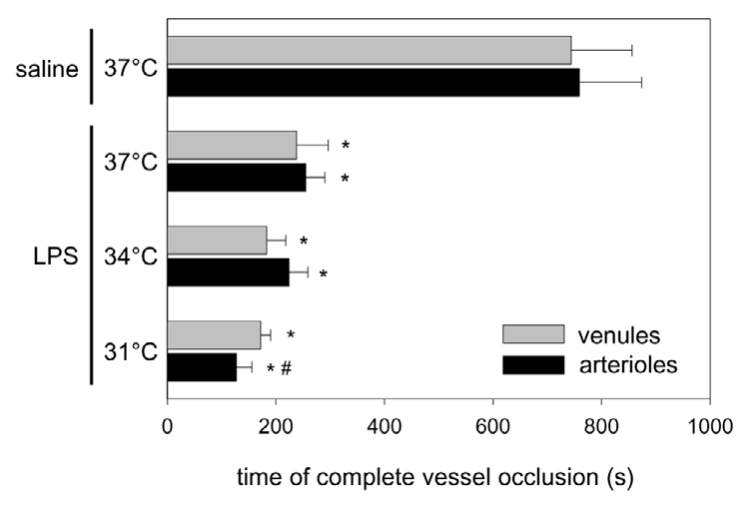
Microvascular thrombus formation *in vivo*. Occlusion times of arterioles and venules upon ferric chloride-induced thrombus formation in 37°C saline controls (10 ml/kg body weight NaCl; -24 h intraperitoneally; N = 10 preparations) and 37°C, 34°C and 31°C endotoxemic animals (10 mg/kg body weight lipopolysacharide (LPS); -24 h intraperitoneally; N = 8 preparations per group). Values are given as means ± standard error of the mean; *p < 0.05 versus 37°C saline controls; ^#^*p* < 0.05 versus 37°C endotoxemic animals.

In saline controls with a body temperature of 37°C, ferric chloride-mediated thrombus formation induced complete occlusion of arterioles and venules after 759 ± 115 s and 744 ± 112 s, respectively (Figure 1). In contrast, in endotoxemic animals, which were maintained at a core body temperature of 37°C, thrombus formation was markedly accelerated, as indicated by significantly reduced arteriolar and venular occlusion times of 255 ± 35 s and 238 ± 58 s, respectively (Figure 1). Systemic hypothermia at 34°C in endotoxemic animals caused a further but only slight and non-significant acceleration of microvascular thrombus formation. Arteriolar and venular vessel lumen were found clogged at an average time of 224 ± 35 s and 183 ± 35 s, respectively.

In both arterioles and venules, continuous cooling of endotoxemic animals to a core body temperature of 31°C resulted in a further acceleration of thrombus formation, in particular in arterioles. While venular occlusion time was found to be decreased only slightly to 172 ± 18 s, arteriolar occlusion time was significantly (*p *< 0.05) reduced (127 ± 29 s) when compared to 37°C endotoxemic controls (Figure 1).

### ELISA of circulating endothelial markers

**Figure 2:**
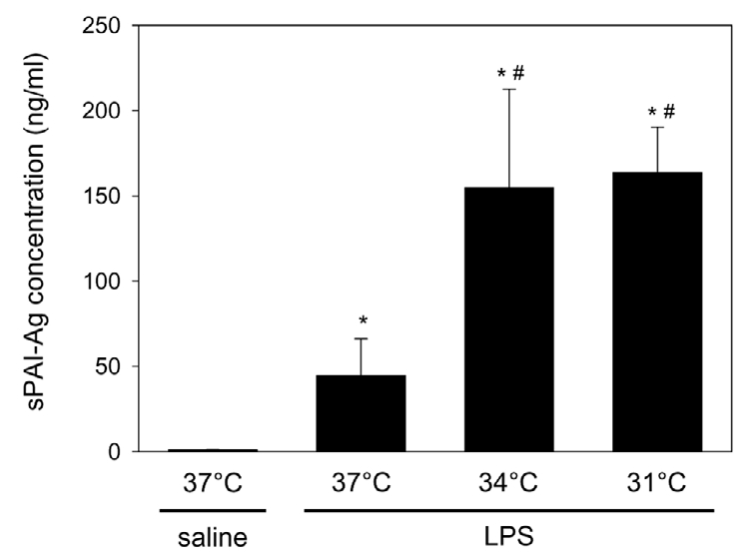
Soluble plasminogen activator inhibitor-1 antigen (sPAI-Ag) concentrations. Plasma concentrations of circulating sPAI-Ag in 37°C saline controls (10 ml/kg body weight NaCl; -24 h intraperitoneally; *n *= 5 animals) and 37°C, 34°C and 31°C endotoxemic animals (10 mg/kg body weight lipopolysacharide (LPS); -24 h intraperitoneally; *n *= 4 animals per group). Values are given as means ± standard error of the mean; *p < 0.05 versus 37°C saline controls; ^#^*p *< 0.05 versus 37°C endotoxemic animals.

To characterize the effect of endotoxemia and hypothermia on endothelial cell activation, we determined circulating (soluble) endothelial activation molecules. In animals with a core body temperature of 37°C, 24 h endotoxemia caused a drastic increase of sPAI-Ag when compared to 37°C saline controls (Figure 2). Of interest, hypothermia of 34°C and 31°C in endotoxemic animals resulted in a further three- to four-fold increase of sPAI-Ag (Figure 2).

**Figure 3:**
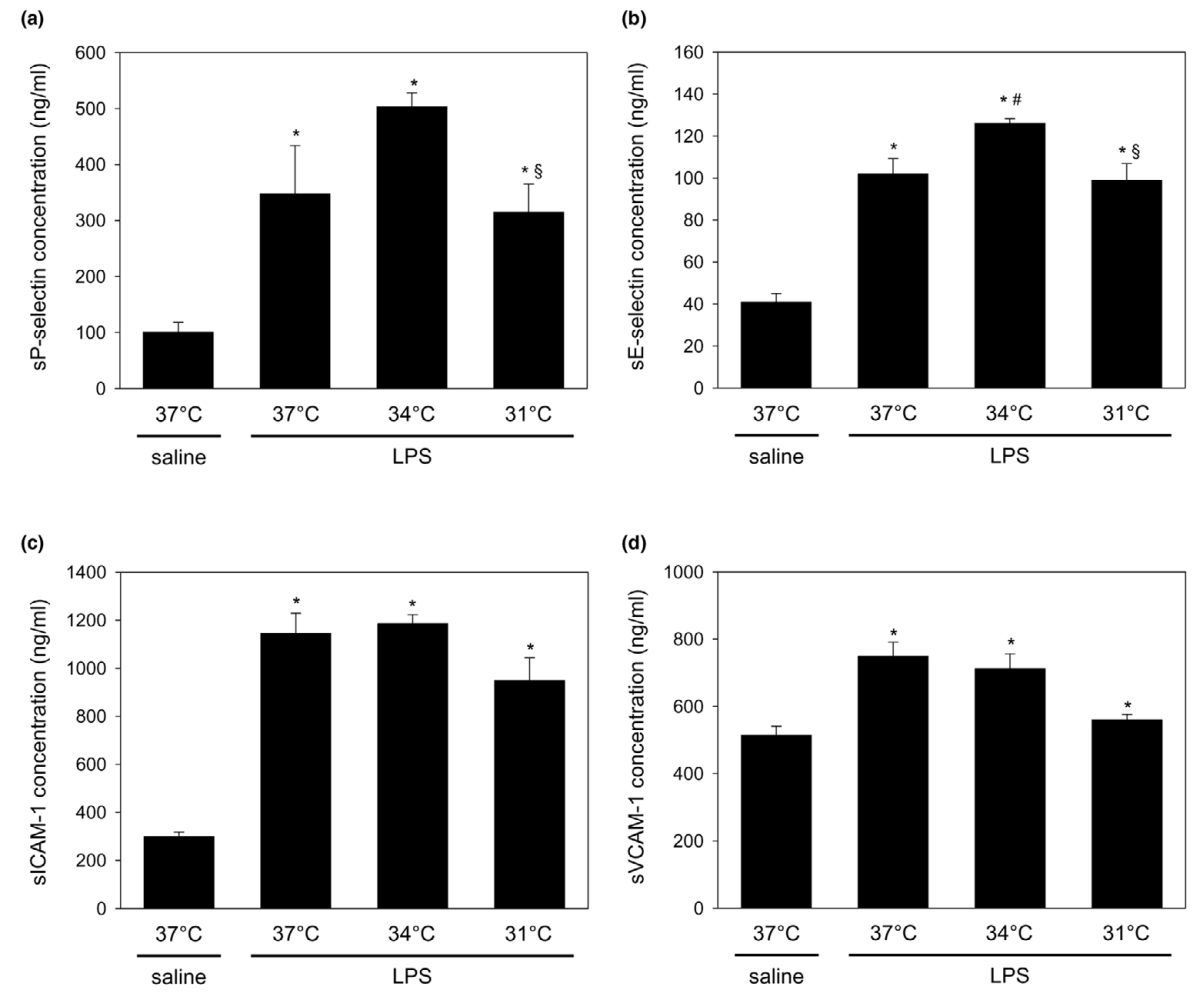
Circulating endothelial activation markers. Plasma concentrations of circulating **(a) **soluble (s)P-selectin, **(b) **sE-selectin, **(c) **intercellular adhesion molecule (sICAM)-1 and **(d) **vascular cell adhesion molecule (sVCAM)-1 in 37°C saline controls (10 ml/kg body weight NaCl; -24 h intraperitoneally; *n *= 5 animals) and 37°C, 34°C and 31°C endotoxemic animals (10 mg/kg body weight lipopolysacharide (LPS); -24 h intraperitoneally; *n *= 4 animals per group). Values are given as means ± standard error of the mean; *p < 0.05 versus 37°C saline controls; ^#^*p *< 0.05 versus 37°C endotoxemic animals; ^§^*p *< 0.05 versus 34°C endotoxemic animals.

In parallel, endotoxemia in 37°C animals induced a marked increase of sP-selectin, sE-selectin, sICAM-1 and sVCAM-1 when compared to 37°C saline controls (Figure 3). However, apart from sE-selectin, these indicators of endothelial activation were not further increased in endotoxemic animals by systemic hypothermia at 34°C and 31°C (Figure 3).

### Flow cytometric analysis of murine platelet P-selectin, glycoprotein IIb-IIIa and CD107a expression

We studied the effect of systemic hypothermia on platelets of LPS-exposed animals. *In vivo *LPS exposure did not significantly affect spontaneous platelet expression of P-selectin, GPIIb-IIIa and CD107a. Also, incubation of platelets from LPS-exposed animals at temperatures of 34°C and 31°C did not result in significant changes in spontaneous P-selectin, GPIIb-IIIa and CD107a expression (data not shown).

In platelets of saline controls (37°C), *in vitro *stimulation with thrombin resulted in elevated expression of P-selectin, GPIIb-IIIa and CD107a. In platelets of endotoxemic 37°C animals, the expression of these markers was slightly, but not significantly, higher compared to saline 37°C warm control animals. However, hypothermic incubation of the LPS-exposed platelets at 34°C and 31°C did not further affect the P-selectin, GPIIb-IIIa and CD107a expression (data not shown).

### Immunohistochemical analysis of P-selectin and PAI-1 expression

**Figure 4:**
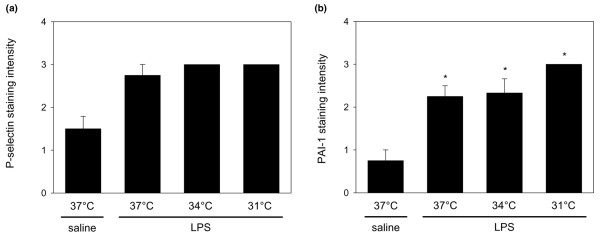
Endothelial P-selectin and plasminogen activator inhibitor (PAI)-1 expression. Analysis of the endothelial expression of **(a) **P-selectin and **(b) **PAI-1 in 37°C saline controls (10 ml/kg body weight NaCl; -24 h intraperitoneally; *n *= 10 tissue specimen) and 37°C, 34°C and 31°C endotoxemic animals (10 mg/kg body weight lipopolysacharide (LPS); -24 h intraperitoneally; *n *= 8 tissue specimens per group). Values are given as means ± standard error of the mean. *p < 0.05 versus 37°C saline controls.

In general, P-selectin and PAI-1 were expressed within the endothelium of arterioles and venules, while little, if any, immunoreactivity was detected within the surrounding muscle tissue. For determination of immunohistological staining, a cross section of the cremaster muscle was evaluated using ×400 magnification. All vessels within this section were assessed, while the total number of vessels did not markedly vary between tissue specimens (20 to 35 vessels with approximately one-third arterioles and two-third venules within each specimen). Endothelial expression of these molecules was assessed by semiquantitative analysis of staining intensity: 0 corresponds to no staining; 1 to faint staining; 2 to moderate staining; and 3 to intense staining. As there were no notable differences in arteriolar and venular endothelial staining, vessels were not differentially assessed. Endotoxemia resulted in a marked increase in the expression of P-selectin and PAI-1 within the microvascular endothelium. In endotoxemic animals endothelial PAI-1 expression was further pronounced by systemic hypothermia at 31°C when compared to animals at 34°C and 37°C (Figure 4a,b).

## Discussion

The major findings of the present study are that LPS-induced endotoxemia is a strong promoter of microvascular thrombosis *in vivo*, most probably due to increased endothelial activation, as indicated by elevated circulating levels of sPAI-Ag, sP-selectin, sE-selectin, sICAM-1 and sVCAM-1. Systemic hypothermia further promotes thrombus formation, particularly in arteriolar vessel structures. Of interest, this hypothermia-induced modulation towards a more procoagulant state is not based on increased expression and release of P-selectin, E-selectin, ICAM-1, VCAM-1 and GPIIb-IIIa because tissue expression and plasma levels of these markers were not affected by the reduction of the core body temperature to 34°C or 31°C. In contrast, the significantly increased sPAI-Ag levels during systemic hypothermia, and the increased endothelial PAI-1 expression in severe hypothermic animals at 31°C may indicate this molecule has a role in aggravation of thrombus formation by low temperatures in endotoxemia.

It is well known that small rodents, mice and rats in particular, initially develop hypothermia after exposure to LPS, which may be followed by a subsequent rise in temperature at later time points [15,16]. The initial hypothermic response seems to be highly dependent on the ambient temperature and the LPS dose [17]. The body temperature usually normalizes after a time period of seven to eight hours, and body rewarming is supposed to be mediated via inducible nitric oxide synthetase [18]. Accordingly, in the present study we observed normothermic temperatures 24 hours after LPS administration.

Previous studies have reported inconsistent data on whether hypothermia affects the expression of surface adhesion molecules on platelets and endothelial cells. *In vitro *hypothermia at 25°C has been shown to inhibit endothelial cell expression of E-selectin [19]. In addition, hypothermic temperatures were found associated with increased P-selectin shedding, although cardiopulmonary bypass patients did not reveal differences in circulating levels of ICAM-1 and VCAM-1 during normothermia and hypothermia [20].

The microvasculature is the critical interface for oxygen and energy delivery to the tissues. Therefore, any obstruction of the microvasculature may have harmful effects on organ function. The generation of pro-inflammatory cytokines during sepsis, including IL-1, IL-6, and IL-8 as well as TNF-alpha activates the endothelial lining cells [21]. The immediate inflammatory response and the stimulation by agonists induce endothelial cell expression of P-selectin. As a result, the surface of the endothelial cells changes from a non-adhesive and non-thrombogenic character towards a pro-adhesive state. In the delayed endothelial response, E-selectin is expressed on endothelial cells after several hours and reaches its maximum after 12 hours [22]. Our results confirm this, in as much as endotoxemia caused a marked rise in shed circulating endothelial markers. To differentiate whether sP-selectin originated from endothelial cells or platelets, which both have been shown to release a soluble form of P-selectin into the plasma [23], we additionally performed immunohistochemical analyses. By this we could show an increased expression of P-selectin in the microvascular endothelium during exdotoxemia, which may indicate that a significant proportion of the circulating sP-selectin originates from the activated endothelium.

To further elucidate the role of platelets, we tested the effect of endotoxemia and systemic hypothermia at 34°C and 31°C on platelet activation and reactivity *in vitro*. Our results indicate that, in addition to the endothelial activation, enhanced platelet reactivity, as caused by the thrombin activation, may contribute to the acceleration of microvascular thrombus formation in endotoxemic animals. Because P-selectin shed from platelets serves as the main source for circulating P-selectin and platelet activation results in up to 50% secretion of intracellular P-selectin [23], it is reasonable to assume that a major part of the increase in sP-selectin during endotoxemia might also be due to platelet activation. Of interest, 34°C and 31°C hypothermia did not further increase spontaneous platelet activation or platelet responsiveness to agonists when compared to normothermic endotoxemic controls. This is most probably due to the fact that endotoxemia already enhanced platelet responsiveness and agonist-induced reactivity, so that little effect could additionally be induced by hypothermia. This view is supported by our previous study, which demonstrated that platelets from healthy humans are highly responsive upon exposure to hypothermic temperatures [8].

Although the importance of GPIb-IX-V in mediating platelet-endothelial interactions is unequivocal, this ligand is thought to be mandatory for adhesion and thrombus growth at high shear [24]. At low shear other adhesion molecules, such as the collagen receptors and GPIIb-IIIa, are mainly involved in platelet adhesion [25,26]. Because the microvessels analyzed in the present study revealed wall shear rates below 300 s^-1^, we elucidated the role of the fibrinogen receptor GPIIb-IIIa. Of interest, spontaneous platelet GPIIb-IIIa expression did not increase but even slightly decreased after endotoxin exposure, and thrombin-stimulation of endotoxin-exposed platelets also induced an only slight but not significant elevation of expression. Because concomitant systemic hypothermia also did not affect GPIIb-IIIa expression, our data suggest that platelet expression of this molecule did not substantively contribute to low temperature-induced acceleration of thrombus formation during endotoxemia.

Although increased levels of plasminogen activators such as tissue plasminogen activator (t-PA) have been observed in sepsis [27], their action appears to be counterbalanced by increased PAI-1 levels, resulting in ineffective fibrinolysis and enhanced organ damage [28]. Recently, it has been recognized that endothelial cells play a pivotal role in the pathogenesis of sepsis by releasing tissue factor thrombomodulin and PAI-1 [2]. For the first time, we now provide evidence that the expression of PAI-1 is increased in the systemic circulation and thrombus formation in endotoxemia is enhanced by moderate systemic hypothermia. This view is supported by the significant increase in circulating PAI-Ag levels at temperatures of 34°C and 31°C versus 37°C in endotoxemic animals, and the most pronounced endothelial expression of PAI-1 during 31°C hypothermia.

Several studies have suggested that PAI-1 plays a major role in the pathogenesis of atherosclerosis and represents a risk factor for coronary heart disease [29]. PAI-1 is the most important physiological inhibitor of tissue plasminogen activator and, therefore, exerts pro-thrombotic effects. APoE-/-mice with high PAI-1 levels exhibit a prothrombotic phenotype with shortened time to thrombotic vessel occlusion in a model of ferric-chloride induced carotid artery injury [30]. The acceleration of thrombus formation observed in endotoxemic and hypothermic animals may, therefore, at least in part, be due to the increase in endothelial PAI-1 expression and plasma concentration. Because hypothermia in general is known to slow down physiological processes, it is possible that hypothermia causes an increase in endothelial PAI-1 expression, while secretion into systemic blood circulation is decelerated or even impaired. This fact might explain why sPAI-1-Ag levels did not increase from 34°C to 31°C, whereas immunohistological staining revealed a further, though not significant, rise in endothelial PAI-1 expression from 34°C to 31°C.

In the pathogenesis of severe coagulation abnormalities in sepsis, three major mechanisms are supposed to play a role: the tissue-factor driven accumulation of thrombin with subsequent fibrinogen conversion, binding to the platelet surface receptor GPIIb-IIIa, and, finally, platelet activation and clotting; impairment of the anti-thrombin, protein C and tissue factor pathway inhibitor anti-coagulative systems; and inhibition of fibrinolysis by increased PAI-1 production [31]. Generally, the increased mortality of hypothermic and septic patients is ascribed to a diminished host response due to an impaired immune function [6,7] and to an augmentation of the generation of inflammatory cytokines like TNF-alpha and IL-1beta [32]. In addition to this, previous studies have shown that correction of hypothermia during sepsis results in decreased IL-6 levels and a significantly increased survival rate [33]. Based on our results, microvascular thrombus formation with the consequence of deterioration of organ perfusion is dramatically increased during the septic state. Although endotoxemia *per se *had already massively reduced microvessel occlusion time, 31°C hypothermia promoted a further, approximately 50% reduction in arteriolar occlusion time, indicating that microvascular thrombus formation may, indeed, at least in part, contribute to the increased mortality rates during systemic hypothermia observed in septic patients.

## Conclusion

Systemic hypothermia superimposed on endotoxemic challenge further increases microvascular thrombus formation *in vivo*. This involves an increase in circulating PAI-1 expression rather than being due to incremental endothelial activation or an elevation of agonist-dependent platelet reactivity.

## Key messages

• LPS-induced endotoxemia accelerates microvascular thrombus formation *in vivo *by generalized endothelial activation and increased platelet reactivity

• Systemic hypothermia further enhances microthrombosis in endotoxemia

• Systemic hypothermia in endotoxemia is associated with increased endothelial PAI-1 expression and sPAI-Ag concentrations

## Abbreviations

bw = body weight; ELISA = enzyme-linked immunosorbent assay; GP = glycoprotein; ICAM = intercellular adhesion molecule; IL = interleukin; ip = intraperitoneally; LPS = lipopolysacharide; PAI = plasminogen activator inhibitor; PAI-1-Ag = plasminogen activator inhibitor-1 antigen; s = soluble; TNF = tumor necrosis factor; VCAM = vascular cell adhesion molecule.

## Competing interests

The authors declare that they have no competing interests.

## Authors' contributions

NL carried out the animal experiments, evaluated the flow cytometric analyses, immunohistological sections and ELISAs, performed the statistics and drafted the manuscript. BV conceived the study, participated in its design and coordination and helped to draft the manuscript. MDM and EK participated in the design and coordination of the study, and in the interpretation of the results. All authors read and approved the final manuscript.

## Figures and Tables

**Table 1:** Red blood cell velocity (μm/s) and wall shear rates (γ; s^-1^) before thrombus formation

	Arterioles		Venules	
	RBC velocity	γ	RBC velocity	γ

Saline-37°C	2200 ± 79	176 ± 21	1720 ± 245	109 ± 11
LPS-37°C	1175 ± 210*	150 ± 29	815 ± 266^a^	124 ± 52
LPS-34°C	1517 ± 203*	188 ± 31	603 ± 72^a^	72 ± 4
LPS-31°C	1063 ± 177*	157 ± 26	662 ± 172^a^	77 ± 29

Thrombus formation was induced by exposure to ferric chloride. Values are given as means ± standard error of the mean. Saline: 37°C saline controls (10 ml/kg body weight NaCl; -24 h intraperitoneally). LPS: endotoxemic animals (10 mg/kg body weight lipopolysaccharide (LPS); -24 h intraperitoneally); 37°C, systemic normothermia; 34°C, 34°C systemic hypothermia; 31°C, 31°C systemic hypothermia. ^a^*p *< 0.05 versus Saline-37°C. RBC, red blood cell.
